# Prevalence and Risk Factors for Esophageal Strictures in Systemic Sclerosis

**DOI:** 10.1002/acr2.90033

**Published:** 2026-04-19

**Authors:** Alannah Quinlivan, Dylan Hansen, Wendy Stevens, Nava Ferdowsi, Susanna Proudman, Jennifer Walker, Joanne Sahhar, Gene‐Siew Ngian, Diane Apostolopoulos, Lauren V. Host, Chamara Basnayake, Kathleen Morrisroe, Laura Ross, Mandana Nikpour

**Affiliations:** ^1^ Department of Rheumatology St Vincent's Hospital Melbourne Victoria Australia; ^2^ Department of Medicine The University of Melbourne at St Vincent's Hospital Melbourne Victoria Australia; ^3^ Department of Gastroenterology St Vincent's Hospital Melbourne Victoria Australia; ^4^ Rheumatology Unit Royal Adelaide Hospital Adelaide South Australia Australia; ^5^ Discipline of Medicine University of Adelaide Adelaide South Australia Australia; ^6^ Rheumatology Unit Flinders Medical Centre Adelaide South Australia Australia; ^7^ Immunology, Allergy and Arthritis Department Flinders University Adelaide South Australia Australia; ^8^ Department of Rheumatology Monash Health Melbourne Victoria Australia; ^9^ Department of Medicine Monash University Melbourne Victoria Australia; ^10^ Department of Rheumatology Fiona Stanley Hospital Perth Western Australia Australia; ^11^ School of Public Health University of Sydney Camperdown New South Wales Australia; ^12^ Department of Rheumatology Royal Prince Alfred Hospital Camperdown New South Wales Australia

## Abstract

**Objective:**

Gastroesophageal reflux disease (GERD) affects up to 90% of patients with systemic sclerosis (SSc). Chronic esophageal acid exposure can result in complications including the formation of esophageal strictures (ES). Proton pump inhibitors can alter gastric acid pH and improve GORD symptoms; however, there have been no recent studies evaluating the prevalence of SSc–ES since these medications became widely available. Our aim was to investigate the prevalence of SSc–ES over time and identify risk factors associated with ES.

**Methods:**

Consecutive patients from the Australian Scleroderma Cohort Study who met American College of Rheumatology/EULAR criteria for SSc were included. Clinically significant ES was defined as characteristic findings seen on gastroscopy or patient‐reported ES requiring dilatation. Multivariable logistic regression analysis was used to identify factors associated with SSc–ES. The time to SSc–ES development from SSc disease onset was evaluated using Kaplan‐Meier survival analysis.

**Results:**

ES affected 191 of 1,543 patients (12.4%) and were associated with a longer disease duration, gastric antral vascular ectasia, esophageal dysmotility, reflux esophagitis, and myocardial disease on multivariable logistic regression analysis (*P* < 0.05). Compared to patients with SSc diagnosed before 1990, those who were diagnosed between 2000 and 2010 and 2010 and 2023 were significantly less likely to have clinically significant ES on multivariable logistic regression analysis (odds ratio 0.45 and 0.42, respectively; *P* = 0.002).The median disease duration at time of SSc–ES diagnosis increased from 3 (interquartile range 0–5) years to 11.5 (interquartile range 3.5–24) years for those diagnosed with SSc–ES before 1990 to those diagnosed after 2010 (*P* = 0.0027).

**Conclusion:**

SSc–ES is associated with a longer disease duration and other gastrointestinal SSc manifestations, with prevalence decreasing over time.

## INTRODUCTION

Systemic sclerosis (SSc) is an autoimmune disease of the connective tissue characterized by autoimmunity, vasculopathy, and fibrosis.^1^ Over 90% of the patients with SSc suffer from upper gastrointestinal (GIT) tract involvement with hallmark features of gastroesophageal reflux disease (GERD), dysmotility, and smooth muscle atrophy.^2^ Patients with SSc are particularly predisposed to GERD due to esophageal dysmotility, a hypotensive lower esophageal sphincter, and delayed gastric emptying.^3^ Erosive esophageal changes can even be seen endoscopically in 77% of the patients with SSc without GIT symptoms less than one year after the SSc diagnosis.^4^ Treatment of GERD in patients with SSc is important to minimize esophageal complications such as esophagitis, Barrett esophagus, and stricture formation.^2^

GERD is the most common cause of esophageal strictures (ES), accounting for 60% to 70% of benign strictures.^5^, ^6^ ES occur following prolonged esophageal acid exposure resulting in inflammation that progresses to fibrosis and loss of esophageal compliance.^5^ ES typically present with progressive dysphagia or solid food dysphagia. The frequency of ES in patients with SSc is up to 15 times that of the general population,^6^, ^7^, ^8^, ^9^ with an estimated prevalence of 17% to 31%. Detection of ES is important because untreated ES can lead to weight loss and malnutrition and, rarely, esophageal perforation.^10^

Proton pump inhibitors (PPIs) decrease the acidity of gastric acid contents, thereby protecting the esophagus from prolonged acid exposure. PPIs have also been shown to decrease ES recurrence following dilatation^6^, ^11^ and, when used for treatment of reflux esophagitis, may prevent the development of ES.^12^, ^13^ These medications have been available in Australia since the early 1990s, with their use increasing^14^ by 1,300% from 1995 to 2006. Previous studies looking at the prevalence of SSc–ES were done before the rise in PPI use. The aim of this study was to determine the frequency of ES in SSc and their associated clinical features and to assess whether a change in frequency of SSc–ES was observed over time.

## PATIENTS AND METHODS

### Study population

All Australian Scleroderma Cohort Study (ASCS) participants who fulfilled the 2013 American College of Rheumatology/EULAR criteria for SSc^15^ were eligible for inclusion in this study. The ASCS is a prospective multicenter cohort study, with data collected annually. Data collected include patient‐reported outcomes, demographics, disease features, medication use, and results of screening tests for pulmonary arterial hypertension (PAH) and interstitial lung disease (ILD) with transthoracic echocardiography and pulmonary function tests (PFTs). Written consent is provided by all the participants before data collection, with ethics approval granted by all human research ethics committees of participating sites.

### Data collection

Demographic data collected included age and sex. Disease‐related data included disease duration (defined as time from onset of the first non‐Raynaud disease manifestation), SSc disease subtype, modified Rodnan skin score, and autoantibody status. Data related to SSc disease complications included ES, ILD, PAH, myocardial disease, myositis, GIT symptoms, reflux esophagitis, Barrett esophagus, esophageal dysmotility, pseudo‐obstruction, small intestinal bacterial overgrowth (SIBO), scleroderma renal crisis, gastric antral vascular ectasia (GAVE), digital ulcers, calcinosis, and joint contractures. The presence of GIT symptoms was recorded as present (yes or no) via annual symptom questionnaire. Clinically significant ES were recorded if characteristic findings were seen on gastroscopy or barium swallow or patients reported a history of ES requiring dilatation. ES was an “ever variable”; therefore, once a patient had been diagnosed with ES, recurrences or clinical improvement postdilatation were not recorded. GIT investigations are performed at the discretion of the treating physician, with indications not recorded in our database. However, common reasons for investigations include refractory reflux symptoms, dysphagia, unexplained anemia, or weight loss. Esophageal dysmotility was defined as definite (with abnormal contractility seen in the high‐resolution manometry [HRM] or barium swallow) or suspected (patient‐reported symptoms of dysphagia to liquids and solids). Reflux esophagitis, Barrett esophagus, and GAVE were recorded if characteristic findings were seen on endoscopy. SIBO was defined as the presence of new diarrhea improved by cyclical antibiotic therapy. Medication use (including PPI, histamine 2 receptor antagonists [H2RA], prednisolone, and nonsteroidal anti‐inflammatory drugs) was recorded present if ever prescribed. ILD was defined by the presence of characteristic pulmonary fibrosis on high‐resolution computed tomography performed in patients with clinical suspicion for ILD (such as abnormalities on PFTs or clinical findings consistent with ILD). PAH was defined by right‐sided heart catheterization according to international criteria.^16^ Myositis was defined as present if all the following criteria were met: (1) muscle weakness on examination, (2) elevated creatine kinase above baseline, and (3) myopathic changes on electromyography or evidence of muscle inflammation on magnetic resonance imaging or muscle biopsy. Myocardial involvement was defined as systolic or diastolic dysfunction or conduction or rhythm abnormalities attributed to SSc by the treating physician.

### Statistical analysis

Data are presented as mean ± SD for normally distributed variables and median (interquartile range [IQR]) for non‐normally distributed continuous variables and as number (percentage) for categorical variables. Differences in frequency were tested using chi‐square test (for categorical variables). For non‐normally distributed continuous variables, the *P* value was calculated using the Kruskal‐Wallis equality‐of‐populations rank test (for three categories) and Wilcoxon rank sum test (for two categories). Multivariable logistic regression analysis was performed using covariates found to be significant on univariate analysis and of clinical relevance. Age was excluded from our multivariable models due to collinearity with disease duration. The relationship between ES and survival was analyzed using Kaplan‐Meier (KM) survival curves and multivariable Cox proportional hazard regression model that included age at disease onset, diffuse disease, PAH, ILD, and sex with the proportional hazards assumption tested using Schoenfeld residuals. The time to SSc–ES development from SSc disease onset was analyzed using KM survival curves. Finally, PPI use over time was examined each year between 2007 (the inception of our cohort) and 2023. A *P* value of <0.05 was considered statistically significant. All statistical analyses were performed using STATA 15.1 (StataCorp).

### Availability of data and materials

The datasets used and/or analyzed during the current study are available from the corresponding author on reasonable request.

## RESULTS

### Patient population

Data from 1,543 participants from the ASCS were included (Table 1). The majority of participants were women (85.3%) with limited disease (74.4%). Participants had a mean age of 43.0 (±31.0) years and a disease duration of 16.5 (±11.2) years. With regards to autoantibody status, 46.1% were centromere positive and 16.0% Scl‐70 positive.

**Table 1:** Demographic and clinical characteristics by ES status*

Characteristics	All patients (n = 1,543)	ES (n = 191)	No strictures (n = 1,352)	*P* value
Age at SSc onset (mean ± SD), years	42.97 (±30.98)	41.36 (±16.09)	43.2 (±32.61)	0.59
Women, n (%)	1,316 (85.3)	174 (91)	1,142 (84.5)	0.01
Disease duration, years	16.46 (±11.2)	21.3 (±12.5)	15.8 (±10.9)	<0.00001
Disease subtype, n (%)				
Limited	1,148 (74.40)	141 (73.82)	1,007 (74.48)	0.85
Diffuse	395 (25.60)	50 (26.2)	345 (25.52)	
ANA centromere (+), n (%)	692 (46.10)	94 (50.8)	598 (45.4)	0.17
Scl‐70 (+), n (%)	238 (16.03)	22 (11.96)	216 (16.6)	0.11
RNA polymerase 3 (+), n (%)	172 (14.39)	26 (16.99)	146 (14.01)	0.33
U1RNP (+), n (%)	95 (6.40)	13 (7.07)	82 (6.31)	0.69
Scl/PM, n (%)	31 (2.1)	1 (0.55)	30 (2.3)	0.12
Highest mRSS^a^, (mean ± SD)	11.55 (±9.77)	11.99 (±8.91)	11.49 (±9.88)	0.51
Joint contractures^a^, n (%)	656 (42.51)	106 (56.1)	550 (41.2)	<0.001
Digital ulcers^a^, n (%)	855 (55.41)	126 (65.97)	729 (53.92)	0.002
Calcinosis^a^, n (%)	130 (10.84)	20 (13.16)	110 (10.51)	0.33
GAVE^a^, n (%)	175 (11.34)	38 (19.9)	137 (10.1)	<0.0001
Reflux esophagitis^a^, n (%)	680 (44.07)	137 (71.73)	543 (40.16)	<0.0001
Esophageal dysmotility symptoms, n (%)	620 (40.18)	149 (78.01)	471 (34.8)	<0.0001
Proven esophageal dysmotility, n (%)	166 (10.8)	47 (24.6)	119 (8.8)	<0.0001
Barrett's esophagus^a^, n (%)	51 (3.31)	8 (4.19)	43 (3.18)	0.47
Pseudo‐obstruction^a^, n (%)	51 (3.31)	11 (5.47)	44 (3.02)	0.11
SIBO^a^, n (%)	134 (8.69)	112 (8.29)	22 (11.52)	0.14
GIT symptoms^a^, n (%)				
Dysphagia	833 (63.44)	156 (77.61)	698 (47.87)	0.0001
Vomiting	400 (25.92)	83(43.68)	317 (23.75)	0.0001
Reflux	1,322 (85.68)	182 (95.29)	1,140 (85.27)	<0.001
Bloating	911 (59.74)	135 (71.05)	776 (58.13)	0.001
Diarrhea	838 (54.84)	128 (67.02)	710 (53.10)	0.0001
Constipation	812 (53.18)	1,294 (65.26)	688 (51.46)	0.0001
Fecal incontinence	526 (34.40)	93 (48.69)	433 (32.36)	0.0001
Myositis^a^, n (%)	113 (7.32)	12 (6.28)	101 (7.47)	0.55
Myocardial disease^a^, n (%)	140 (9.07)	29 (15.18)	111 (8.21)	0.002
Renal crisis^a^, n (%)	57 (3.69)	5 (2.62)	52 (3.85)	0.40
PAH, n (%)	190 (12.31)	31 (16.23)	159 (11.76)	0.07
ILD, n (%)	520 (33.7)	63 (32.98)	457 (33.8)	0.82
GORD treatment^a^, n (%)				
PPI use	1,301 (84.32)	1,586 (97.38)	1,115 (82.47)	0.0001
H2RA use	364 (23.61)	78 (40.84)	286 (21.17)	0.0001
Promotility agents^a^, n (%)	237 (15.37)	59 (30.89)	178 (13.18)	0.0001
Prednisolone^a^, n (%)	667 (43.23)	94 (49.21)	573 (42.38)	0.07
NSAID use^a^, n (%)	521 (33.77)	75 (39.27)	446 (32.99)	0.09
Date of SSc diagnosis, n (%)				
<1990	227 (15.6)	51 (27.7)	176 (13.9)	<0.0001
1991–2000	294 (20.21)	50 (27.2)	244 (19.2)	
2001–2010	554 (38.08)	57 (30.98)	497 (39.1)	
2011–2023	380 (25.74)	26 (14.13)	354 (27.85)	

* PAH defined as mean pulmonary artery pressure ≥20 mm Hg and a pulmonary capillary wedge pressure ≤15 mm Hg and pulmonary vascular resistance ≥ 2 Woods units on right‐sided heart catheter. ILD is defined as the presence of characteristic pulmonary fibrosis on high‐resolution computed tomography of the chest. ANA, antinuclear antibody; GAVE, gastric antral vascular ectasia; GIT, gastrointestinal; GORD, gastroesophageal reflux disease; H2RA, histamine 2 receptor antagonist; ILD, interstitial lung disease; mRSS, modified Rodnan skin score; NSAID, nonsteroidal anti‐inflammatory drug; OS, esophageal strictures; PAH, pulmonary arterial hypertension; SSc, systemic sclerosis; SIBO, small intestinal bacterial overgrowth.

^a^ Ever recorded during follow‐up.

### ES

ES were recorded in 191 of 1,543 of participants (12.4%). On univariate analysis, female sex, disease duration, presence of joint contractures, GAVE, esophageal dysmotility, digital ulcers, reflux esophagitis, and myocardial disease were significantly associated with ES (*P* < 0.05; Table 1). Additionally, participants with ES were more likely to report GIT symptoms throughout the whole GIT tract in addition to symptoms of reflux and dysphagia, including vomiting, bloating, diarrhea, constipation, and fecal incontinence (*P* < 0.001).

On multivariable logistic regression analysis, disease duration (odds ratio [OR] 1.03, *P* < 0.0001), GAVE (OR 1.61, *P* = 0.04), myocardial disease (OR 1.92, *P* = 0.008), symptoms of esophageal dysmotility (OR 5.11, *P* < 0.0001), definite esophageal dysmotility (OR 2.06, *P* = 0.001), and reflux esophagitis (OR 1.95, *P* < 0.0001) remained significantly associated with clinically significant ES (Table 2). When looking at patient‐reported GIT symptoms, only dysphagia (OR 2.86, *P* < 0.0001) was significantly associated with a history of ES (Supplementary Table 1).

**Table 2:** Multivariable logistic regression model of demographic and disease features associated with systemic sclerosis esophageal strictures*

Variable	OR	95% CI	*P* value
Women	1.56	0.89–2.72	0.12
Disease duration	1.03	1.02–1.05	<0.0001
GAVE^a^	1.61	1.02–2.54	0.04
Esophageal dysmotility symptoms^a^	5.11	3.45–7.57	<0.0001
Proven esophageal dysmotility	2.06	1.36–3.12	0.001
Reflux esophagitis^a^	1.95	1.34–2.81	<0.0001
Myocardial disease^a^	1.92	1.19–3.13	0.008
Digital ulcers^a^	1.06	0.73–1.54	0.76
Joint contractures^a^	1.29	0.90–1.84	0.17

* CI, confidence interval; GAVE, gastric antral vascular ectasia; OR, odds ratio.

^a^ Ever recorded during follow‐up.

Overall, 1,301 patients (84.3%) were ever prescribed a PPI and 364 (23.6%) were prescribed an H2RA. Of those who had ever been prescribed a PPI, 91.9% (1,195 of 1,301) were documented to be on a PPI at their most recent visit. There was no significant difference in rates of PPI and H2RA prescription between those with and without ES (Supplementary Table 1). The use of PPI in our cohort did not increase over time, with 71.6% of participants prescribed a PPI in 2007 and 71.7% prescribed a PPI in 2023.

### ES over time

The proportion of patients with SSc with clinically significant ES declined over time from 22.5% for those diagnosed before 1990, 17% diagnosed between 1990 and 2000, 10.3% diagnosed between 2000 and 2010, to 6.8% diagnosed after 2010. Compared to patients with SSc diagnosed before 1990, those who were diagnosed between 2000 and 2010 and 2010 and 2023 were significantly less likely to suffer ES on multivariable logistic regression analysis (OR 0.45 and 0.42, respectively; *P* < 0.001; Supplementary Table 2).

**Figure 1:**
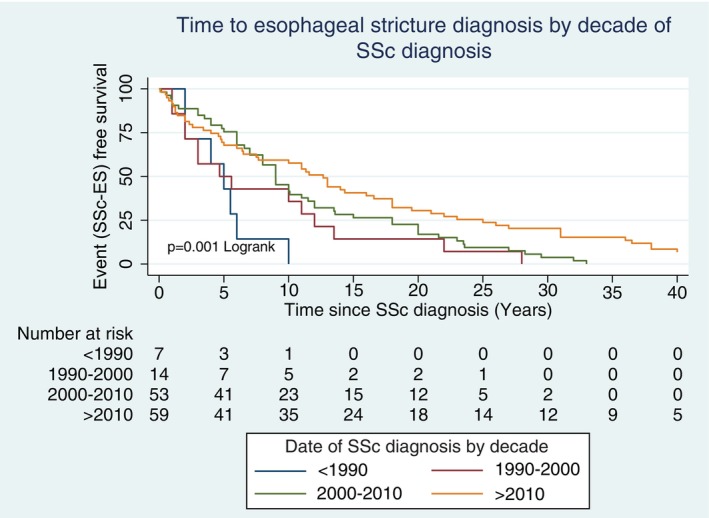
Time to ES diagnosis by decade. ES, esophageal strictures; RP, Raynaud phenomenon; SSc, systemic sclerosis.

The date of ES diagnosis was available for 160 of 191 (83.8%) patients, of which 12 (7.5%) were diagnosed before 1990, 18 (11.3%) between 1990 and 2000, 62 (38.8%) between 2000 and 2010, and 68 (42.5%) between 2010 and 2023 (see Supplementary Table 3). The number of new diagnoses of ES after enrollment in the ASCS (64 of 160) has been stable at a median of 3 (IQR 2–6) participants per year across all sites since 2007. At the time of ES diagnosis, additional findings recorded in our database included reflux esophagitis in 151 of 160 (94.4%), GAVE in 14 of 160 (8.9%), and Barrett's esophagus in 2 of 160 (1.3%). The median disease duration before development of clinically significant SSc–ES was 8 years (IQR 2–18) with the median age at diagnosis 54.5 (IQR 46.5–65) years. Over time, the median disease duration at the time of SSc–ES diagnosis increased from 3 (IQR 0–5) years to 11.5 (IQR 3.5–24) years for those diagnosed with SSc–ES before 1990 to those diagnosed after 2010 (*P* = 0.0027; see Figure 1). The median age at SSc–ES diagnosis also increased from 37 (IQR 31–46) years to 60 (IQR 52–70.5) years (*P* = 0.0001), whereas the median age at SSc diagnosis was not significantly different between the groups (*P* = 0.22).

### ES and death

**Figure 2:**
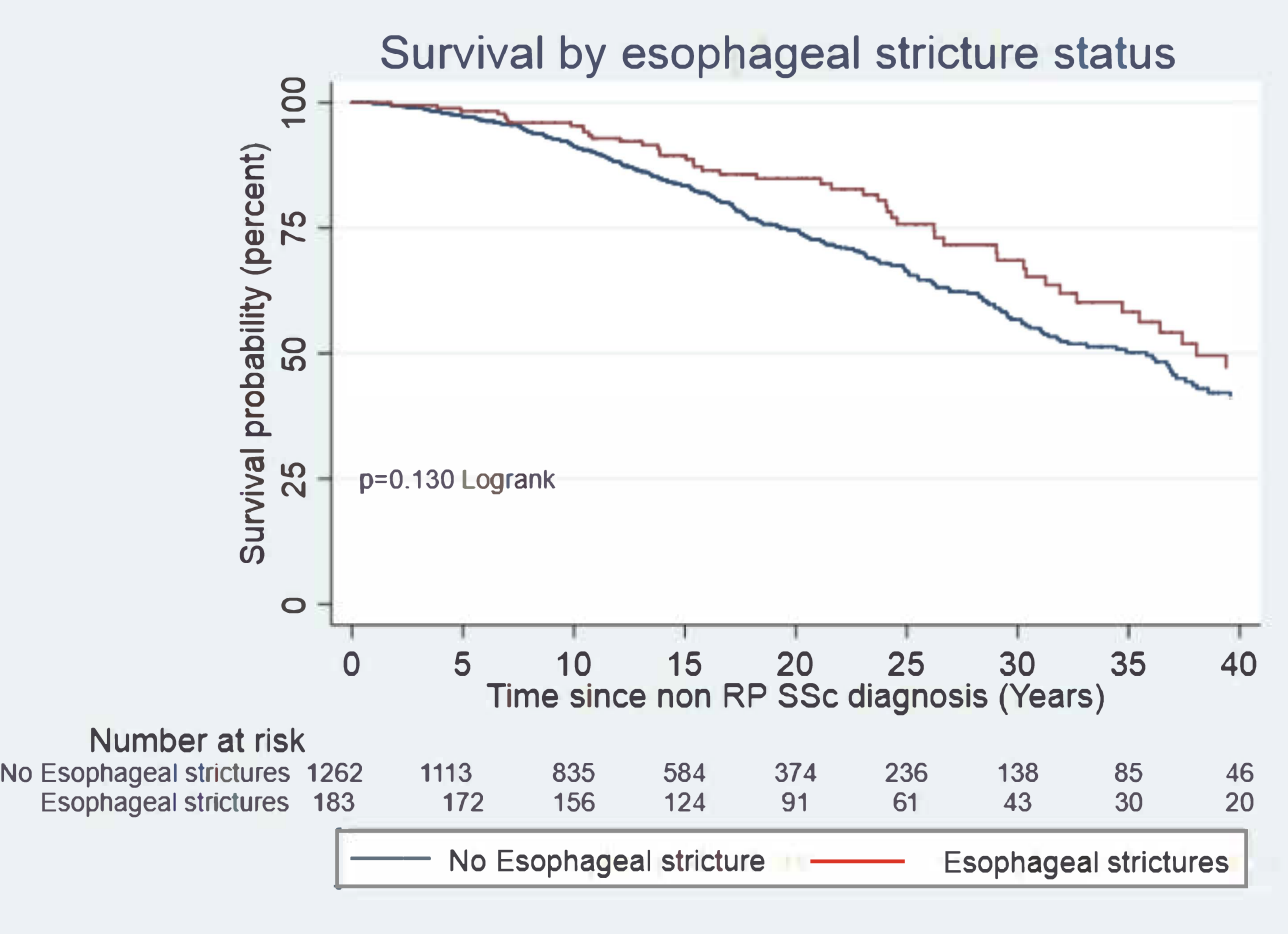
Survival by esophageal stricture status. RP, Raynaud phenomenon; SSc, systemic sclerosis.

In total, 374 patients died during follow‐up, of whom 58 had ES. There was no significant difference with regard to mortality rates between participants with ES compared to those without (*P* = 0.13; Figure 2). Hazard regression analysis confirmed there was no association between ES and death (hazard ratio [HR] 0.97, *P* = 0.81; Table 3).

**Table 3:** Multivariable Cox proportional hazard regression model for survival from disease onset according to ES status*

Variable	OR	95% CI	*P* value
ES	0.97	0.73–1.29	0.81
Women	0.56	0.44–0.73	<0.0001
Diffuse SSc	1.96	1.54–2.51	<0.0001
PAH^a^	2.05	1.64–2.56	<0.0001
ILD^a^	1.65	1.33–2.04	<0.0001
Age at disease onset	1.10	1.10–1.11	<0.0001

* PAH defined as mean pulmonary artery pressure ≥20 mm Hg and a pulmonary capillary wedge pressure ≤15 mm Hg and pulmonary vascular resistance ≥ 2 Woods units on right‐sided heart catheter. ILD defined as the presence of characteristic pulmonary fibrosis on high‐resolution computed tomography of the chest. CI, confidence interval; ILD, interstitial lung disease; ES, esophageal strictures; OR, odds ratio; PAH, pulmonary arterial hypertension; SSc, systemic sclerosis.

^a^ Ever recorded during follow‐up.

## DISCUSSION

ES were present in 12.4% of our SSc cohort. The prevalence of ES in the ASCS was lower than previously reported rates of ES (ranging from 17% to 25% in unselected patients)^3^, ^9^, ^17^, ^18^, ^19^ and rates of ES appear to be decreasing over time. Previous studies conducted before 1990 showed a prevalence^17^, ^18^, ^19^ of 25%, which decreased to 17% from a study published in 1994.^3^ This is in keeping with our findings, which show a prevalence of 22.5% for those diagnosed before 1990 and 17% for those diagnosed between 1990 and 2000. We also found that patients diagnosed between 2000 and 2010 had a prevalence of 10.3%; however, it is important to note that this patient group may not yet have had sufficient time to develop ES because our data suggest that in a contemporary treatment era, ES develop more than 10 years after SSc disease onset.

Factors significantly associated with ES included longer disease duration, GAVE, myocardial disease, reflux esophagitis, and esophageal dysmotility. Because the pathogenesis of ES formation involves prolonged esophageal exposure to gastric acid (in part due to esophageal dysmotility) resulting in inflammation (reflux esophagitis), the association we found between these variables and ES formation is logical.^5^ Additionally, factors diagnosed on endoscopy (such as GAVE and reflux esophagitis) may be more commonly found in those undergoing gastroscopy for other reasons. It is notable that we observed an association between ES and GAVE and myocardial disease. The pathophysiology of GIT dysmotility, GAVE, and myocardial disease has been suggested to be secondary to immune‐mediated vasculopathy, which may explain the association among these manifestations.^20^ With regard to GAVE, our findings are in keeping with a previous study showing the association between GAVE and GIT dysmotility.^20^ When looking at the risk factors for ES for the general population, these include older age (>60 years), symptoms of reflux and dysphagia, reflux symptoms of more than six months duration, and esophagitis.^21^, ^22^ Although our patients had a lower median age at SSc–ES diagnosis (54.5 years, IQR 46.5–65.0), over time this is becoming closer to the general population, with the median age for SSc–ES diagnosed after 2010 being 60 years (IQR 52.0–70.5).

Participants with SSc–ES were more likely to report GIT symptoms affecting all areas of the GIT tract on univariate analysis; however, it did not reach significance on multivariable analysis. Although >85% of the patients with ES in our cohort reported symptoms of reflux, this symptom was not significantly associated with ES, which may be due to the low number of patients who reported no history of reflux.

In Australia, PPI use increased dramatically until 2007, with 84.3% of participants in our cohort having been exposed to PPIs. The majority of patients with SSc–ES were diagnosed with SSc before 2000, with ~28% diagnosed before 1990. Those diagnosed with SSc between 2000 and 2010 and 2010 and 2023 had a reduced risk of ES compared to those diagnosed before 1990 (OR 0.45 and 0.42, respectively; *P* < 0.002). Patients diagnosed with SSc before 2000 are likely to have experienced many years of reflux before PPIs became widely available.^14^, ^23^ There is an absence of prospective clinical trials that have evaluated the impact of PPIs on ES. However, PPIs have been shown to reduce recurrence of ES following dilatation (HR 0.6) compared with both no treatment and H2RA prescription.^6^, ^11^, ^24^, ^25^, ^26^ Furthermore, large population‐based studies from the United States have shown that the rates of ES requiring dilatation decreased between 1994 and 2000, which corresponds to the increased use of PPIs in the United States.^27^, ^28^

The median disease duration before development of SSc–ES was eight years (95% confidence interval [CI] 2–18); therefore, this result may indicate that those diagnosed with SSc after 2010 have not had sufficient time for SSc–ES to develop. However, our data also revealed that, over time, the disease duration at SSc–ES diagnosis increased significantly from 3 (CI 0–5) to 11.5 (CI 3.5–24) years for those diagnosed with SSc–ES before 1990 and after 2010, respectively (*P* = 0.0027). Because the majority of our patients were diagnosed with SSc–ES before enrollment in our cohort, we are unable to determine the earlier PPI use. However, the prevalence of SSc–ES has decreased since use of these agents has become more widespread in Australia.

There was no association between SSc–ES and death in our cohort, which is in keeping with data from the general population.^6^ To our knowledge, there have been no other studies in SSc examining the association between SSc–ES and survival because the previous studies were cross‐sectional or had very short follow‐up periods.^3^, ^17^, ^18^, ^19^ The lack of association found in our cohort may, in part, be due to survival bias. Because the majority of strictures were diagnosed before initiation of the ASCS database, this may select for patients with more stable disease. Another possible explanation is that strictures are treatable with dilatation and therefore may not lead to severe GIT complications such as malnutrition known to be associated with reduced survival.

Our study has a number of strengths, including up to date data from a large number of well‐characterized patients with SSc. To our knowledge, this is the first study to detail risk factors associated with SSc–ES in SSc and the only study since 1998 to detail the prevalence of SSc–ES. However, our study is not without limitations. Many participants were diagnosed with SSc–ES before entry into the cohort (127 of 191, 66.5%), and because our cohort was established in 2007, data pertaining to those diagnosed with SSc–ES between 1990 and 2000 are limited. Because not all patients underwent gastroscopy, those with mild reflux symptoms or minimal strictures may have been missed; therefore, we may have underestimated the prevalence of strictures. Additionally, not all patients underwent HRM; therefore, our prevalence of esophageal dysmotility may also be an underestimation. We also do not have detailed data relating to the additional findings on gastroscopy but assumed the documented findings of reflux esophagitis, Barrett esophagus, and GAVE were present because they were diagnosed the same year as ES. Data pertaining to PPI use or clinical features (such as reflux) before enrollment in the ASCS were not available. Additionally, data regarding specific PPI used, dose of PPI, or medication compliance are not collected in the ASCS. We were also unable to perform time‐varying analysis because the date of ES diagnosis typically occurred before entry into the cohort.

## CONCLUSION

Although ES are more common in SSc than the general population, their frequency is decreasing over time with an overall estimated prevalence of 12.4% corresponding to the increased rates of PPI usage.

## AUTHOR CONTRIBUTIONS

All authors contributed to at least one of the following manuscript preparation roles: conceptualization AND/OR methodology, software, investigation, formal analysis, data curation, visualization, and validation AND drafting or reviewing/editing the final draft. As corresponding author, Prof Nikpour confirms that all authors have provided the final approval of the version to be published and takes responsibility for the affirmations regarding article submission (eg, not under consideration by another journal), the integrity of the data presented, and the statements regarding compliance with institutional review board or Declaration of Helsinki requirements.

## Supporting information


**Disclosure Form**:

**Supplementary Table 1.** Multivariable logistic regression model of features associated with OS on univariate logistic regression analysis.
**Supplementary Table 2**. Multivariable logistic regression model of OS risk factors according to epoch of diagnosis.
**Supplementary Table 3**. Age and disease duration at SSc‐OS diagnosis according to epochs.
