# Supplementary material for: Prevalence and Risk Factors for Esophageal Strictures in Systemic Sclerosis
Source: ACR Open Rheumatol. 2026 Apr 19;8(4):e90033. doi: 10.1002/acr2.90033 (PMC13092343; doi:10.1002/acr2.90033)
Supplement: Supplementary file 2 — Supplementary Table 1. Multivariable logistic regression model of features associated with OS on univariate logistic regression analysis. Supplementary Table 2. Multivariable logistic regression model of OS risk factors according to epoch of diagnosis. Supplementary Table 3. Age and disease duration at SSc‐OS diagnosis according to epochs. [file ACR2-8-e90033-s001.docx]

Supplementary Table 1- Multivariable logistic regression model of features associated with ES on univariate logistic regression analysis.

| **Variable** | **OR** | **95% CI** | **value** |
| --- | --- | --- | --- |
| Female | 1.42 | 0.77-2.59 | 0.26 |
| Disease duration | 1.03 | 1.02-1.05 | <0.0001 |
| GAVE* | 1.79 | 1.12-2.88 | 0.02 |
| Oesophageal dysmotility* | 3.70 | 2.39-5.73 | <0.0001 |
| Dysphagia* | 2.86 | 1.60-5.23 | <0.0001 |
| Reflux* | 1.78 | 0.82-3.85 | 0.14 |
| Vomiting* | 1.39 | 0.98-1.98 | 0.06 |
| Bloating* | 1.04 | 0.72-1.52 | 0.83 |
| Diarrhoea* | 1.07 | 0.75-1.54 | 0.71 |
| Constipation* | 1.19 | 0.83-1.69 | 0.35 |
| Faecal incontinence* | 1.08 | 0.76-1.53 | 0.43 |
| Myocardial disease* | 1.87 | 1.13-3.09 | 0.02 |
| Digital ulcers* | 0.95 | 0.63-1.42 | 0.79 |
| Joint contractures* | 1.24 | 0.84-1.83 | 0.27 |
| PPI* | 1.78 | 0.61-5.20 | 0.29 |
| H2RA* | 1.13 | 0.77-1.68 | 0.53 |
| Promotility agent* | 1.32 | 0.87-2.00 | 0.19 |

*Abbrev: SSc- systemic sclerosis ES- esophageal stricture, OR- odds ratio, CI- confidence interval, GAVE- Gastric antral vascular ectasia, PPI- proton pump inhibitor, H2RA- histamine 2 receptor antagonist, PPI- proton pump inhibitor, H2RA- histamine 2 receptor antagonist*

*Ever recorded during follow up

Supplementary Table 2 - Multivariable logistic regression model of ES risk factors according to epoch of diagnosis

| **Variable** | **OR** | **95% CI** | | **p value** | |
| --- | --- | --- | --- | --- | --- |
| Female gender | 1.57 | 0.90-2.74 | | 0.11 | |
| Date of SSc diagnosis |  |  |  | |  |
| <1990 | 1.00 |  | |  | |
| 1990-2000 | 0.71 | 0.44-1.15 | | 0.16 | |
| 2000-2010 | 0.45 | 0.29-0.71 | | 0.001 | |
| 2010-2023 | 0.42 | 0.25-0.73 | | 0.002 | |
| GAVE* | 1.60 | 1.01-2.53 | | 0.04 | |
| Oesophageal dysmotility* | 5.05 | 3.42-7.47 | | <0.0001 | |
| Reflux oesophagitis* | 1.91 | 1.32-2.78 | | 0.001 | |
| Myocardial disease* | 1.86 | 1.15-3.03 | | 0.012 | |
| Digital ulcers | 1.08 | 0.74-1.57 | | 0.70 | |
| Joint contractures | 1.23 | 0.86-1.77 | | 0.25 | |

*Abbrev: SSc- systemic sclerosis ES- esophageal stricture, OR- odds ratio, CI- confidence interval, GAVE- Gastric antral vascular ectasia, PPI- proton pump inhibitor, H2RA- histamine 2 receptor antagonist*

**Ever recorded during follow up*

Supplementary Table 3 – Age and disease duration at SSc-ES diagnosis according to epochs

| **SSc-ES epoch of diagnosis** | **All SSc-ES (n=160)** | **<1990**  **(n=12)** | **1991-2000**  **(n=18)** | **2001-2010**  **(n=62)** | **>2010**  **(n=68)** | **p value** |
| --- | --- | --- | --- | --- | --- | --- |
| Age at diagnosis*  (Median, IQR) | 54.5 (46.5-65) | 37  (31-46) | 49  (44-55) | 54  (48-65) | 60  (52-70.5) | 0.0001 |
|  |  |  |  |  |  |  |
| Disease duration at diagnosis* (Median, IQR) | 8  (2-18) | 3  (0-5) | 3  (1-11) | 9  (4-15) | 11.5  (3.5-24) | 0.003 |
|  |  |  |  |  |  |  |
| Age at SSc diagnosis*  (Median, IQR) | 43  (34.5-55) | 37.5 (30.5-44.5) | 44.5  (33-54) | 43.5  (35-55) | 45  (36.5-57.5) | 0.22 |

* in years

*SSc; systemic sclerosis, ES; esophageal stricture, n; number, IQR; interquartile range*
